# Local Anæsthesia—Employment of Hardy’s Instrument

**Published:** 1854-04

**Authors:** 


*Local Anæsthesia—Employment of Hardy's Instrument.*

The first essay with this instrument was made by M. Nelaton, at the Clinique, upon a girl who was suffering from abscess of the axilla, and from a small wound upon the back of the wrist, both extremely painful. The instrument was composed of a caoutchouc reservoir of air adapted to a copper pump, made to receive the sponge for the chloroform. A valve at one extremity permitted the air to enter the instrument, which terminated in a caoutchouc tube. The first application of the chloroform upon the tumour of the axilla produced an insensibility which lasted three hours, during which time the part could be handled and examined with impunity. In the second essay, M. Dubois plunged a knife into the abscess, which was ripe, after the employment of the chloroformic fumigation. The patient declared that she was not conscious of pain, and became aware of the fact that the abscess was opened only by touching the part with her hand. From this time she had no more pain. The little wound on the wrist, fumigated in the same manner, remained quite insensible.—*Ibid.*

